# 15-Year trends, predictors, and outcomes of heart failure hospitalization complicating first acute myocardial infarction in the modern percutaneous coronary intervention era

**DOI:** 10.1093/ehjopen/oeaf013

**Published:** 2025-02-19

**Authors:** Muhammad Rashid, Dmitry Abramov, Muhammad Usman Naseer, Harriette G C Van Spall, Fozia Z Ahmed, Claire Lawson, Mohamed Dafaalla, Evangelos Kontopantelis, Mohamed O Mohamed, Mark C Petrie, Mamas A Mamas

**Affiliations:** Department of Cardiovascular Sciences, University of Leicester and the National Institute for Health Research Leicester Biomedical Research Centre, Glenfield Hospital, LE1 7RH Leicester, UK; Department of Cardiology, Glenfield Hospital, University Hospitals of Leicester, LE3 9QP Leicester, UK; Keele Cardiovascular Research Group, School of Medicine, Keele University, ST5 5BG, UK; Division of Cardiology, Loma Linda University Medical Center, Loma Linda, CA 92354, USA; Department of Cardiology, Glenfield Hospital, University Hospitals of Leicester, LE3 9QP Leicester, UK; Department of Medicine, McMaster University, Hamilton, ON, Canada L8S 4L8; Department of Cardiology, Manchester University Hospitals NHS Foundation Trust, M13 9WL Manchester, UK; Department of Cardiovascular Sciences, University of Leicester and the National Institute for Health Research Leicester Biomedical Research Centre, Glenfield Hospital, LE1 7RH Leicester, UK; Keele Cardiovascular Research Group, School of Medicine, Keele University, ST5 5BG, UK; Division of Informatics, Imaging and Data Science, Faculty of Biology, Medicine and Health, University of Manchester, M13 9NT Manchester, UK; Institute of Health Informatics, University College, NW1 2DA London, UK; BHF Cardiovascular Research Centre, University of Glasgow, G12 8TA Glasgow, UK; Keele Cardiovascular Research Group, School of Medicine, Keele University, ST5 5BG, UK

**Keywords:** Heart failure, Acute myocardial infarction, Trends, Risk factors, Mortality

## Abstract

**Aims:**

Heart failure (HF) following acute myocardial infarction (AMI) is a global health concern, but data on risk factors associated with HF hospitalization post-AMI are limited.

**Methods and results:**

We analysed data from the Myocardial Ischaemia National Audit Project, including patients admitted with AMI from 1 January 2006 to 31 March 2019. Data linkage with Hospital Episode Statistics Admitted Patient Care and the Office for National Statistics facilitated a longitudinal analysis. High-risk patients were identified using dapagliflozin in patients without diabetes mellitus with acute myocardial infarction (DAPA-MI) and EMPAgliflozin on Hospitalization for Heart Failure and Mortality in Patients With aCuTe Myocardial Infarction (EMPACT-MI) criteria. We assessed clinical outcomes, adherence to European Society of Cardiology quality indicators, and predictors of HF-related hospitalizations. Out of 1 046 480 AMI patients, 9.1% overall, 17.2% in the DAPA-MI cohort, and 16.6% in the EMPACT-MI cohort experienced HF hospitalization within a year post-AMI. High-risk patients, defined by the presence of five risk factors, had nearly one in four hospitalizations with HF at 1-year follow-up. The predicted adjusted incidence rate for heart failure within 1 year almost doubled from 64.5 cases per 1000 person-years [95% confidence interval (CI): 51.1 to 78.0] in 2005, to 118.2 cases per 1000 person-years in 2019 (95% CI: 115.0 to 121.5). Heart failure hospitalization was associated with a three-fold increase in 1-year mortality (hazard ratio 3.01, 95% CI 2.95–3.13).

**Conclusion:**

One in 10 AMI patients experienced HF hospitalization within the first-year post-AMI, with rising trends in high-risk subgroups. These findings highlight the need for targeted post-AMI care strategies to improve outcomes and address the increasing burden of HF in the modern percutaneous coronary intervention era.

## Graphical abstract

**Graphical Abstract:**
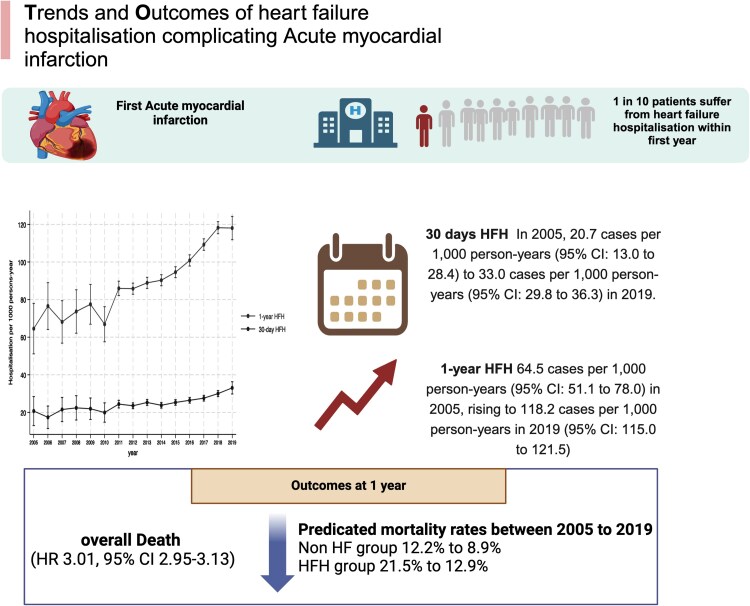

## Introduction

Heart failure (HF) is a global health problem, affecting an estimated 56 million people worldwide^1,2^ and is the leading cause of hospitalization in the USA and across Europe.^2–4^ Acute myocardial infarction (AMI) is the most common aetiology of HF.^5^ While advances in pharmacological and interventional therapies have resulted in better survival rates following AMI, patients remain at significant risk of subsequent cardiovascular events, particularly the development of HF.^6–9^

Multiple epidemiological studies have described opposing trajectories in the incidence, secular trends, and clinical outcomes of HF following AMI.^6,7,9–12^ A national report from Scotland reported declining trends in HF-related hospitalization (HFH) over the past 25 years.^11^ Another analysis of the National Readmission Database showed that related HF-related hospitalizations declined from 2010 to 2014, followed by an increase from 2014 to 2017.^12^ Notably, the literature on the association of known clinical risk factors such as age, diabetes, and previous history of AMI^13,14^ or implementation of guideline-directed therapies^15^ during the index AMI admission with subsequent HFH is lacking. EMPAgliflozin on Hospitalization for Heart Failure and Mortality in Patients With aCuTe Myocardial Infarction (EMPACT-MI)^16^ and dapagliflozin in patients without diabetes mellitus with acute myocardial infarction (DAPA-MI)^17^ are trials that tested the efficacy of SGLT2i in study populations that were enriched for risk for CV death or HFH by including at least one additional cardiovascular risk factor.

This study aims to examine the incidence and trends of 30-day and 1-year HFH in a national cohort of patients admitted with AMI. To elucidate the epidemiological relationship between known risk factors for HF development and their subsequent association with HFH, we analysed HFH rates utilizing the inclusion criteria from the DAPA-MI and EMPACT-MI trials. Additionally, we explored the association between HFH and 1-year mortality.

## Methods

### Study setting

Myocardial Ischemia National Audit Project (MINAP) is the UK heart attack registry enrolling patients admitted with a diagnosis of AMI to any of the 230 acute National Health Service (NHS) hospitals.^18,19^ MINAP collects information about patient demographics, cardiovascular comorbidities, pre-hospital, in-hospital and post-discharge medications, other relevant investigations, and guidelines indicated treatment administered.^20–22^ The post-discharge information was obtained by linking the MINAP with Hospital Episode Statistics Admitted Patient Care (HES-APC) database. Hospital Episode Statistics Admitted Patient Care is an administrative database which collects information about all hospital admissions in England. Finally, the mortality outcomes were obtained from the Office of National Statistics (ONS), the official national death registry of the UK.^23^Myocardial Ischemia National Audit Project registry was linked using patient identifiers in the form of a unique NHS number, date of birth, and patient postcode with the HES-APC and ONS dataset to create the longitudinal data for this study. The study underwent formal ethical approval for the data linkages of the MINAP, HES, and ONS registries. Ethical approval was granted by the Health and Care Research Wales (HCRW) and the Health Research Authority (HRA, Research Ethics Committee reference 20/WA/0312). Additionally, approval was obtained by the Confidentiality Advisory Group (CAG), an independent body providing expert advice on the use of confidential patient information for research. Data may be requested from the National Institute for Cardiovascular Outcomes Research, https://www.nicor.org.uk/. Further details on data request applications may be found at https://www.nicor.org.uk/national-cardiac-audit-programme/heart-attackaudit-minap.

### Study population

The study cohort comprised all patients admitted with a diagnosis of AMI in the MINAP registry between 1 January 2006 and 31 March 2019. We only included the index AMI admission in a 12-month period in patients with multiple admissions. The NHS identifier and the date of subsequent hospitalizations from the HES-APC database were used to identify the occurrence and date of the first hospitalization with acute heart failure using the ICD-10-CM codes (I11.0, I13.0, I13.2, I50.1, I50.2, I50.3, I50.4, I50.8, I50.9) in the primary diagnosis field. Any patients with missing NHS identifiers, age, and discharge date were excluded from the analysis. For patients with more than one hospitalization after the index AMI admission, only the first hospitalization with acute heart failure diagnosis in the HES-APC was included in the analysis. The final study cohort was divided into non-HFH and HFH group. The European Society of Cardiology (ESC) Association for Acute Cardiovascular Care quality indicators (QIs) related to AMI were used to evaluate the quality of care provided during the index AMI admission and whether it is associated with increased risk of HFH.^15^ These indicators included the prescription of angiotensin-converting enzyme inhibitors or angiotensin receptor blockers (ACEI/ARB), beta-blockers, dual antiplatelets, and high dose statin on discharge, reperfusion within 12 h after the presentation for ST-elevation acute myocardial infarction (STEMI), door to balloon time, revascularization [percutaneous coronary intervention (PCI)/coronary artery bypass grafting (CABG)], left ventricular ejection fraction (LVEF) evaluation prior to discharge, invasive coronary angiography within 24 h for non-ST-elevation myocardial infarction (NSTEMI), use of high sensitive troponin assay for diagnosis of NSTEMI, in-hospital LDL-C assessment, and parenteral anticoagulation administration during in-hospital admission.^15^

Patients with high-risk CV risk features were defined as those meeting EMPACT-MI and DAPA-MI inclusion criteria such as symptoms of HF on admission defined Kilip Classes II–IV, LVEF < 45%, age >65 years, prior AMI, eGFR < 60 mL/min/1.73 m^2^, Type 2 DM, patients not revascularized after the AMI, and peripheral vascular disease.

Ethical approval for the data linkages using patient-identifiable information was provided by the HCRW and the HRA (REC reference 20/WA/0312). It was also approved by CAG, an independent body that provides expert advice on using confidential patient information for research. However, no patient-identifiable information was shared with the research or analytical team.^24–27^

### Clinical outcomes

The primary clinical outcome was hospitalization with HF in patients who survived to discharge at 30 days and 1 year after the index AMI. The secondary outcome included 1-year mortality within 1 year of AMI among those with an HFH event. Temporal trends in rates of HFH and independent predictors of HFH at 1 year were measured. To understand the rates of first HFH in clinical trial populations, analyses were undertaken based on the eligibility criteria of DAPA-MI and EMPACT-MI trials (see Supplementary material online, *Table S1*). To address survivor bias, a landmark analysis was performed, excluding patients who died within the first year after an HFH event.

#### Statistical analyses

Continuous data were reported as mean and standard deviation or as the median and interquartile range if the data were non-uniformly distributed. Categorical variables were expressed using percentages and compared using the *χ*^2^ test, and the *t*-test were used to test for statistical significance between categorical and continuous variables, respectively. The Kruskal–Wallis test was used for non-uniformly distributed variables. The cumulative incidence of HFH in the overall population, stratified according to diabetes, sex, and age, was estimated using the Kaplan–Meier estimate. Missing data were accounted for using multiple imputations with chained equations, assuming that data were missing at random, and 10 imputed data sets were generated.^28–30^ All subsequent analyses were performed on the imputed dataset. Cox proportional hazard regression models were used to calculate hazard ratios (HRs) for 1-year mortality and HFH after adjusting for potential confounding factors. We used an interaction term in the indicator variable (heart failure hospitalization) and year of admission as an ordinal variable to estimate the odds of 1 year mortality. Margins command in STATA was used to produce predicted estimates of mortality per year. We constructed Poisson regression models, including HFH within 30 days and 1 year as an outcome variable and age, gender, ethnicity, cardiac arrest, cardiogenic shock, LVEF, history of angina, previous MI, diabetes (DM), hypertension, hypercholesterolaemia, peripheral vascular disease, stroke, family history (FH) of CAD, smoking, chronic kidney disease, asthma/chronic obstructive airway disease (COPD), previous PCI, and previous CABG and year as covariates to calculate incidence rate ratios of HFH with 95% confidence intervals (95% CI). Post-estimation commands were used to estimate absolute rates of HFH within 30 days and 1 year per 1000 person-years per calendar year. Fine Grey competing risk regression models were used to identify the independent predictors of HFH accounting for competing risk of death. The variables adjusted for in the models were age, gender, ethnicity, cardiac arrest, cardiogenic shock, LVEF, history of angina, previous MI, diabetes (DM), hypertension, hypercholesterolaemia, peripheral vascular disease, stroke, FH of CAD, smoking, chronic kidney disease, asthma/COPD, previous PCI, previous CABG and discharge medication including dual antiplatelet medication, statins, beta-blocker, ACEI and ARBs, and aldosterone antagonists. Model estimates were reported as HR and 95% CI. The STATA V16 software was used to perform the statistical analysis.

## Results

The study population comprised 1 046 480 patients admitted with AMI in the MINAP registry from 1st January 2006 to 31st March 2019, out of which 95 281 (9.1%) patients were admitted with heart failure within the first year of the index admission (*Table 1* and Supplementary material online, *Figure S1*).

**Table 1:** Characteristics of patients without and with a HF hospitalization at 1 year

	Overall	No HF hospitalization at 1 year *n* (%)	HF hospitalization at 1 year *n* (%)	*P*-value
*n*	1 046 480	951 199 (90.9)	95 281 (9.1)	
Age at admission, mean (SD)	1 046 480	68.8 (13.8)	76.2 (12.3)	<0.001
Women	353 425	317 662 (33.5)	35 437 (37.2)	<0.001
Ethnicity	936 024			<0.001
White		769 505 (90.6)	79 569 (9.4)	
BAME		78 138 (89.9)	89.87 (10.1)	
BMI (kg/m^2^), mean (SD)	458 104	27.1 (6.9)	27.6 (7.1)	<0.001
High-risk characteristics for HF hospitalization				
Killip class				<0.001
Killip Class I	377 187	348 847 (78.2)	28 378 (58.1)	
Killip Class II	91 817	78 379 (17.6)	13 477 (27.7)	
Killip Class III	21 312	15 134 (3.4)	6187 (27.7)	
Killip Class IV	4236	3450 (0.8)	791 (1.6)	
Good LV function	254 327	240 828 (59.7)	13 489 (28.4)	
Moderate/poor LV function	196 200	162 322 (40.3)	33 941 (71.5)	<0.001
Diabetes	1 004 700	193 598 (21.2)	32 255 (35.0)	<0.001
Peripheral vascular disease	934 717	35 798 (4.2)	7259 (8.5)	<0.001
Non-invasive strategy	1 002 885	343 761 (40.4)	46 982 (54.9)	<0.001
Age > 65 years	633 633	559 446 (58.8)	74 187 (77.9)	<0.001
eGFR < 60 mL/min/1.73 m^2^	241 680	203 315 (27.9)	38 365 (50.4)	<0.001
Comorbidities				
Angina	271 158	235 388 (27.2)	35 777 (41.0)	<0.001
Previous heart failure	57 789	42 148 (4.9)	15 641 (18.1)	<0.001
Hypertension	496 727	443 675 (50.7)	53 052 (60.2)	<0.001
Hypercholesterolaemia	332 792	300 140 (35.1)	32 652 (38.0)	<0.001
Cerebrovascular disease	78 336	66 946 (7.8)	11 390 (13.2)	<0.001
Smoking status				<0.001
Never smoked	372 677	337 924 (38.3)	34 753 (39.7)	
Ex-smoker	335 404	299 828 (34.1)	35 576 (40.6)	
Current smoker	259 765	242 490 (27.5)	17 275 (19.7)	
Asthma/COPD	147 877	129 397 (15.1)	18 480 (21.5)	<0.001
**ESC quality of care indicators**				
Seen by a cardiologist	597 660	546 465 (59.2)	51 295 (55.4)	<0.001
ACE on discharge	774 692	702 282 (76.6)	72 410 (78.4)	<0.001
Beta-blocker on discharge	760 988	689 582 (75.4)	71 406 (77.5)	<0.001
Statin on discharge	872 020	790 307 (86.4)	81 713 (88.5)	<0.001
DAPT on discharge	972 932	883 445 (94.6)	89 487 (95.0)	<0.001
In-hospital LDL-C measurement	613 258	563 129 (97.0)	50 129 (96.1)	<0.001
LV assessment in hospital	538 028	481 934 (67.4)	56 094 (74.5)	<0.001
P2Y12 use in hospital	835 962	759 184 (84.3)	76 778 (84.1)	0.05
Pre-hospital ECG	201 883	187 079 (70.7)	14 804 (69.7)	0.002
Reperfusion for STEMI	269 240	251 151 (82.3)	18 089 (74.9)	<0.001
Timely reperfusion for STEMI	159 166	149 654 (49.1)	9512 (39.4)	<0.001
hc-TnI for NSTEMI	556 651	497 250 (98.4)	59 401 (98.7)	<0.001
Parenteral anticoagulation	625 783	566 856 (59.6)	58 927 (61.8)	<0.001
In patient PCI	288 375	270 846 (37.4)	17 529 (24.9)	<0.001
Inpatient CABG	21 830	20 223 (2.8)	1607 (2.3)	<0.001
30 day mortality	18 444	16 758 (1.7)	1686 (1.7%)	0.937
1-year mortality	115 965	91 563 (9.6)	24 402 (25.6)	<0.001

BAME, British Asian Minority Ethnic; BMI, body mass index; non-invasive strategy, patient not receiving coronary angiogram/PCI or CABG; COPD, chronic obstructive airway disease; DAPT, dual antiplatelet therapy; ECG, electrocardiogram; STEMI, ST-elevation acute myocardial infarction; NSTEMI, non-ST-elevation acute myocardial infarction; hc-TNI, high sensitivity troponin I assay; PCI, percutaneous coronary intervention; CABG, coronary artery bypass grafting.

Patients in the HFH group were almost 7 years older than the non-HFH group (76.2 years vs. 68.8 years), were more likely to be women (37.2% vs. 33.5%) and had a previous history of smoking (40.6% vs. 34.1%). Patients in the HFH group also had a higher prevalence of comorbidities, including pre-existing heart failure, angina, stroke, asthma or COPD, and hypertension. The assessment of risk factors defined by EMPACT and DAPA-MI inclusion criteria revealed a higher prevalence of Killip Classes II–IV, left ventricle systolic dysfunction, diabetes, peripheral vascular disease, and renal disease defined eGFR < 60 mL/min/1.73 m^2^ in the HFH group. Patients in the cohort who did not have a HFH were more likely to be seen by a cardiologist (59.2% vs. 55.4%), receive timely reperfusion in the form of primary PCI (82.3% vs. 74.9%) and had higher rates of revascularization in the form of PCI (37.4% vs. 24.9%) and CABG (2.9% vs. 2.3%). The HFH group were more likely to be discharged on appropriate guidelines recommended pharmacology in the form of beta-blockers (77.5% vs. 75.4%), angiotensin inhibitors or receptor blockers (ACE/ARB) (78.4% vs. 76.6%), high-intensity statin (88.5% vs. 86.5%), and dual antiplatelet therapy (95% vs. 94.6%).

The study characteristics after excluding patients with known previous heart failure are reported in *Table 2*, showing a similar demographic and risk profile compared to the overall study population.

**Table 2:** Baseline characteristics after excluding those with previous chronic heart failure diagnosis

	Overall *n* (%)	No HF hospitalization at 1 year	HF hospitalization at 1 year	*P*-value
*n*	886 697	816 013 (92)	70 684 (8.0)	
Age at admission, mean (SD)	886 697	68.2 (13.8)	75.7 (12.3)	<0.001
Women	296 011 (33.3)	270 094 (33.1)	25 917 (36.6)	<0.001
Ethnicity				<0.001
White	717 123 (90.6)	658 293 (90.7)	58 830 (8.2)	
BAME	73 965 (9.4)	67 452 (91.2)	6513 (8.8)	
BMI (kg/m^2^), mean (SD)		27.1 (6.9)	27.6 (7.2)	<0.001
Risk factors for HF hospitalization				
Killip class				<0.001
Killip Class I	338 954 (78.1)	316 213 (79.6)	22 741 (61.2)	<0.001
Killip Class II	76 418 (17.6)	66 860 (16.8)	9558 (25.7)	
Killip Class III	15 168 (3.5)	10 933 (2.7)	4235 (11.4)	
Killip Class IV	3411 (0.8)	2784 (0.7%)	627 (1.7%)	
Good LV function	233 105 (58.7)	138 380 (61.6)	11 152 (30.2)	<0.001
Moderate/poor LV function	164 175 (41.3)	138 380 (38.4)	25 795 (69.8)	<0.001
Diabetes	187 389 (21.4)	164 117 (20.4)	23 272 (33.3)	<0.001
Peripheral vascular disease	35 952 (4.1)	30 735 (3.8)	5217 (7.5)	<0.001
Non-invasive strategy	276 413 (32.4)	245 490 (31.2)	30 923 (45.7)	<0.001
Age > 65 years	524 892 (59.2)	470 708 (57.7)	54 184 (76.6)	<0.001
eGFR < 60 mL/min/1.73 m^2^	197 903 (28)	170 503 (26.2)	27 400 (47.2)	<0.001
Comorbidities				
Angina	231 548 (26.3)	205 757 (25.3)	25 827 (36.8)	<0.001
Hypertension	443 102 (50.2)	401 899 (49.5)	41 203 (58.8)	<0.001
Hypercholesterolaemia	299 684 (34.5)	274 557 (34.4)	25 127 (36.3)	<0.001
Cerebrovascular disease	67 101 (7.6)	58 591 (7.2)	8727 (12.3%)	<0.001
Smoking status				<0.001
Never smoked	322 697 (38.2)	296 448 (38.1)	26 249 (39.4)	
Ex-smoker	288 318 (34.1)	262 726 (28.1)	26 229 (39.4)	
Current smoker	232 787 (27.6)	218 726 (28.1)	14 061 (21.1)	
Asthma/COPD	129 341 (14.8)	115 644 (14.4)	13 697 (19.6)	<0.001

	Overall	All AMI	HF hospitalization	*P*-value
**ESC quality of care indicators**				
Admitted by a cardiologist	516 714 (59.1)	477 286 (59.3)	39 428 (56.4)	<0.001
ACE on discharge	671 539 (77.3)	616 805 (77.1)	54 734 (79%)	<0.001
Beta-blocker on discharge	658 021 (75.9)	604 271 (75.8)	43 750 (77.7)	<0.001
Statin on discharge	695 072 (93.9)	639 081 (93.9)	55 991 (93.9)	0.94
DAPT on discharge	838 845 (95.2)	771 482 (95.2)	67 363 (97.7)	<0.001
In-hospital LDL-C measurement	546 976 (97.0)	507 258 (97.1)	39 718 (96.4)	<0.001
LV assessment in hospital	474 934 (68.0)	430 815 (67.3)	44 119 (75.5)	<0.001
P2Y12 use in hospital	680 314 (88.8)	625 981 (88.9)	54 333 (88.7)	0.397
Pre-hospital ECG	181 426 (70.5)	168 824 (70.6)	12 422 (69.4)	0.001
Reperfusion for STEMI	230 100 (82.1)	215 375 (82.6)	14 725 (75.7)	<0.001
Timely reperfusion for STEMI	140 404 (50.1)	132 331 (50.7)	8073 (41.5)	<0.001
hc-TnI for NSTEMI	474 174 (98.4)	431 123 (98.4)	43 051 (98.6)	<0.001
Parenteral anticoagulation	571 974 (64.5)	525 149 (64.3)	46 825 (66.2)	<0.001
In patient PCI	256 673 (36.7)	242 840 (38)	13 833 (25.5)	<0.001
In-patient CABG	19 463 (2.8)	18 121 (2.8)	1342 (2.5)	<0.001
30 days mortality	13 970 (1.6)	12 738 (1.5)	1232 (1.7)	<0.001
1-year mortality	87 351 (9.8)	70 289 (8.6)	17 062 (24.1)	<0.001

BAME, British Asian Minority Ethnic; BMI, body mass index; non-invasive strategy, patient not receiving coronary angiogram/PCI or CABG; COPD, chronic obstructive airway disease; ECG, electrocardiogram; STEMI, ST-elevation acute myocardial infarction; NSTEMI, non-ST-elevation acute myocardial infarction; hc-TNI, high sensitivity troponin I assay; PCI, percutaneous coronary intervention; CABG, coronary artery bypass grafting.

### Rates, trends, and predictors of heart failure hospitalizations

**Figure 1:**
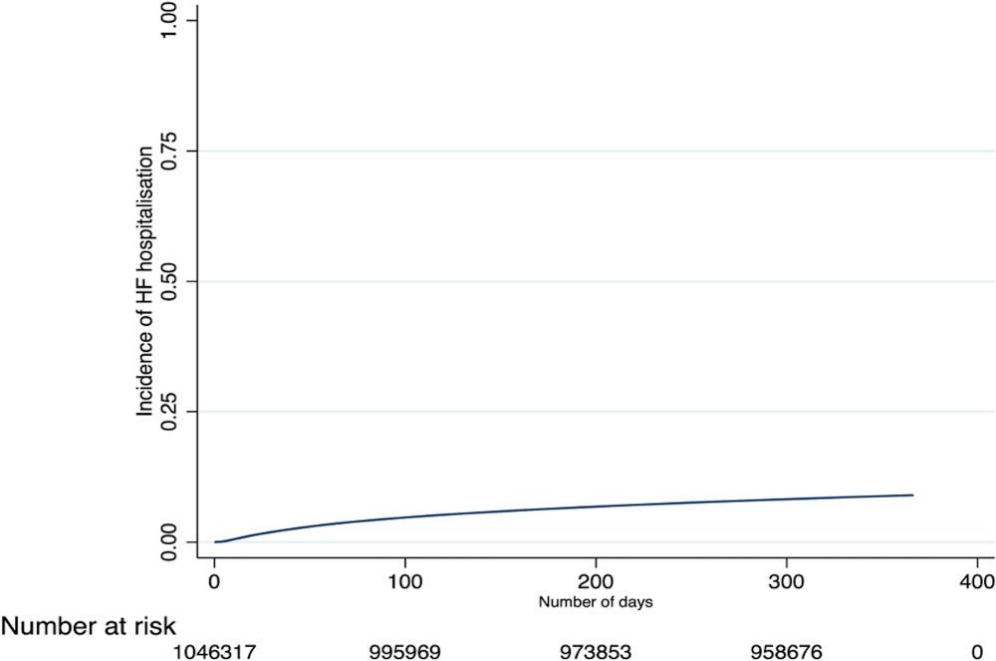
Cumulative incidence of first heart failure hospitalization over 1 year following index acute myocardial infarction.

**Figure 2:**
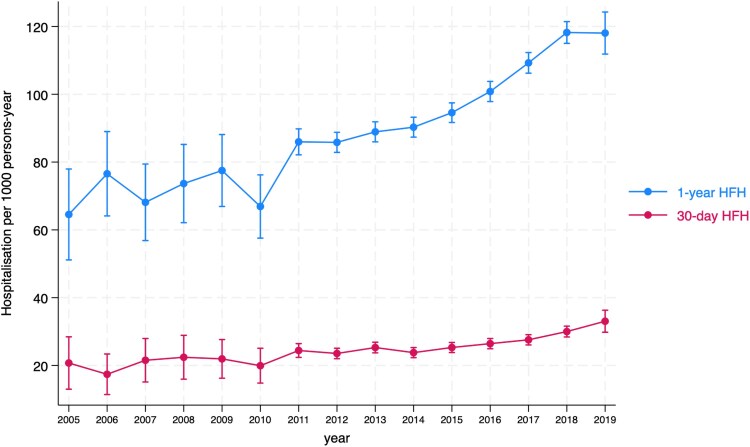
Temporal trends in predicated rates of heart failure hospitalizations at 30 days and 1 year during the study period.

There was a steady increase in the cumulative incidence of HFH, with 1-year hospitalization rates of 9.1% (*Figure 1*). In 2005, the predicted adjusted incidence rate for heart failure within 30 days was 20.7 cases per 1000 person-years (95% CI: 13.0 to 28.4). In 2019, this rate increased to 33.0 cases per 1000 person-years (95% CI: 29.8 to 36.3). Similarly, the predicted adjusted incidence rate for heart failure within 1 year in 2005 was 64.5 cases per 1000 person-years (95% CI: 51.1 to 78.0), rising to 118.2 cases per 1000 person-years in 2019 (95% CI: 115.0 to 121.5) (*Figure 2*). The HFH rates at 30 days rose from 2.3% in 2006 to 3.2% in 2019, and at 1 year from 8.0% in 2006 to 11.5% in 2019 (see Supplementary material online, *Figure S2*). Subgroup analysis revealed the 1-year incidence of HFH was almost doubled in patients with a history of diabetes compared to those without diabetes (14.4% vs. 7.7%) (see Supplementary material online, *Figure S3*). A similar increasing trend was observed in HFH rates among female and older patients (see Supplementary material online, *Figures S4* and *S5*).

**Figure 3:**
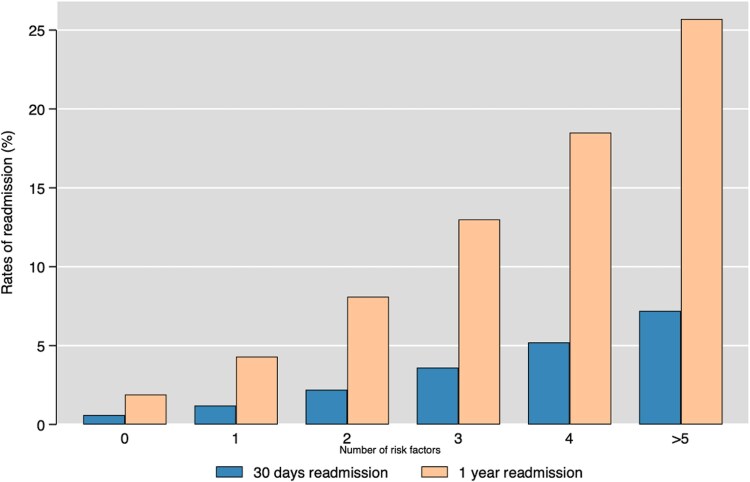
Rates of 30-day and 1-year heart failure hospitalizations stratified according to the number of risk factors for heart failure hospitalizations defined by EMPACT-MI trial.

**Figure 4:**
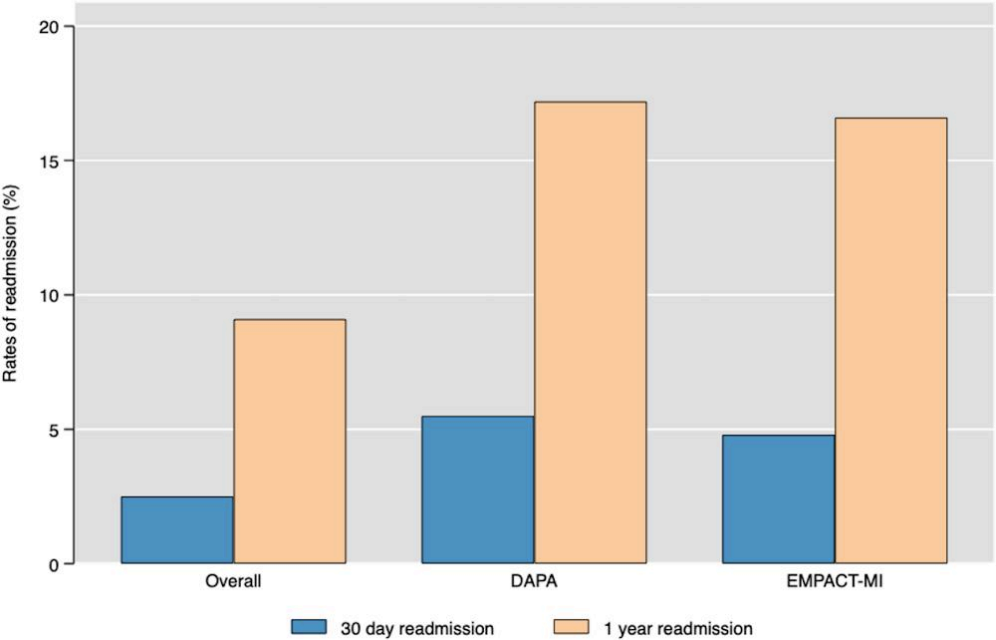
Rates of 30-day and 1-year hospitalization meeting eligibility criteria of EMPACT-MI, DAPA-MI trial.

The 30-day and 1-year HFH rates in patients meeting the inclusion criteria for the EMPACT-MI trial were notably higher, with the highest rates at 30 days (7.2%) and 1 year (25.7%) seen in patients with five or more risk factors as defined by the EMPACT-MI trial (*Figure 3*). Compared to the overall cohort, patients eligible for the DAPA-MI and EMPACT-MI trials exhibited higher rates of HFH at 30 days (DAPA-MI 5.5%, EMPACT-MI 4.8%) and 1 year (DAPA-MI 17.2%, EMPACT-MI 16.6%), respectively (*Figure 4*).

The independent predictors of 1-year HF-related hospitalizations are detailed in *Table 3*. Notably, increasing age per year (HR 1.0004 [1.002–1.005]), female sex (HR 1.04 [1.03–1.06]), moderate left ventricular (LV) dysfunction at index AMI (HR 1.61 [1.56–1.67]), severe LV dysfunction at index AMI (HR 2.46 [2.37–2.56]), and the number of risk factors for HF hospitalization as defined by the EMPACT-MI trial were associated with higher 1-year hospitalization rates (*Table 3*).

**Table 3:** Independent predictors of 1-year HF hospitalization

Independent predictors	Subdistribution HR (95% CI)
Age (per year)	1.0004 (1.002–1.005)
Female sex	1.04 (1.03–1.06)
Moderate LV dysfunction	1.61 (1.56–1.67)
Severe LV dysfunction	2.46 (2.37–2.56)
Previous AMI	1.27 (1.23–1.31)
Diabetes mellitus	1.06 (1.03–1.09)
Hypertension	1.04 (1.01–1.06)
Cerebrovascular disease	1.12 (1.08–1.16)
Asthma or COPD	1.21 (1.17–1.24)
Kilip class II	1.09 (1.05–1.23)
Kilip class III	1.50 (1.44–1.56)
Kilip Class IV	1.99 (1.91.2.13)
Number of risk factor as inclusion criteria of EMPACT trial	
One risk factor	2.15 (1.98–2.34)
Two risk factor	3.85 (3.54–4.19)
Three risk factors	5.84 (5.34–6.38)
Four risk factors	7.59 (6.91–8.35)
Five or more risk factors	8.87 (7.99–9.84)

**Figure 5:**
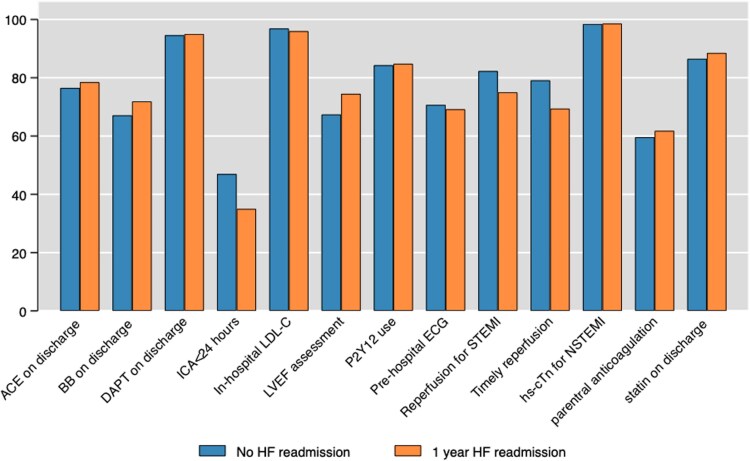
Rates of 30-day and 1-year hospitalization stratified according to European Society of Cardiology quality indicators.

Finally, stratification of patients according to the ESC quality indicators demonstrated that timely reperfusion or any reperfusion in STEMI and the use of early invasive coronary angiography in NSTEMI patients were associated with lower 1-year HFH rates (*Figure 5*).

### Mortality outcomes

**Figure 6:**
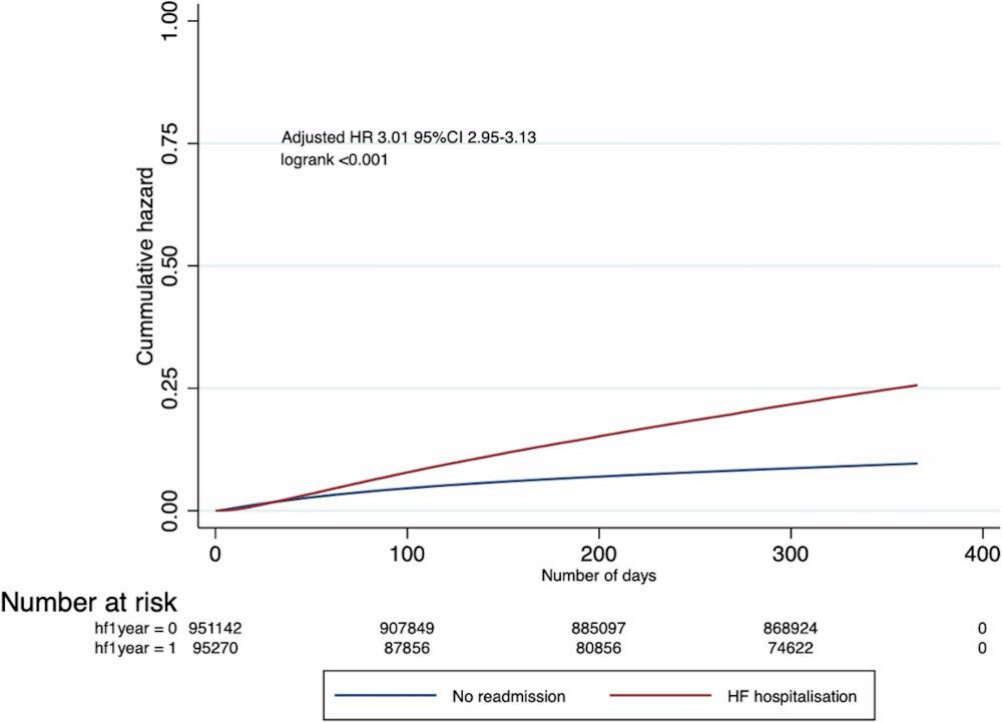
1-year mortality in patients admitted with heart failure within 1 year after acute myocardial infarction compared to those without heart failure hospitalization.

The crude rates of 30-day mortality were similar in both groups with and without a HF hospitalization (1.7% vs. 1.7%), whereas 1-year mortality was significantly higher in the HFH group compared to the non-HFH group (25.6% vs. 9.6%). After adjustment of all available potential confounders, time to HFH within a year of AMI was associated with an almost three-fold increased hazard of 1-year mortality [HR 3.01 95% CI (2.95–3.13)] (*Figure 6*). The landmark analysis after excluding the patients who died within first year of index AMI admission showed increased hazard of 1 year mortality [HR 1.28 95% CI (1.25–132)] in patients with HFH group compared to non-HFH group. Over the study period, there was a notable decline in the adjusted probability of 1-year mortality following AMI, irrespective of the presence of acute HF within the first year. In the non-HF group, the predicted probability of 1-year mortality decreased significantly from 12.2% in 2005 to 8.9% in 2019. A similar trend was observed in those with HFH, where mortality rates fell from 21.5% to 12.9%.

## Discussion

In this national population-based study of over 1 million patients admitted with AMI in the contemporary revascularization era, approximately 1 in 10 patients were re-admitted with acute HF within the first-year post-discharge. The cumulative number of risk factors, as defined by EMPACT-MI and DAPA-MI randomized controlled trials, was associated with up to a six-fold increase in acute HFH. The secular trends analysis over almost 15 years depicted rising trends in 30-day and 1-year HFH rates, particularly in the subgroups with diabetes, female sex, and those over 80 years. HFH was associated with increased hazard of 1 year adjusted mortality at subsequent follow-up.

Previous epidemiological investigations have reported heterogeneous trends in the incidence of HF hospitalization following an AMI.^9–12^ A Medicare fee-for-service beneficiaries database analysis from 1998 to 2010 showed a modest reduction in 1-year HF hospitalization rates, decreasing from 16.1 per 100 person-years in 1998 to 14.2 per 100 person-years in 2010.^10^ Similarly, in Scotland, the incidence of HF hospitalization following a first AMI exhibited a decreasing trend from 1991 to 2016, suggesting a positive impact of improved AMI management and secondary prevention strategies on HF risk at the population level.^11^ Conversely, a nationwide analysis, including 86 771 Patients from the Cardiovascular Disease in Norway Project, reported HF admission rates of 32.6% at 1 year following the index AMI.^31^ In the present analysis from a contemporary cohort of AMI patients in the PCI era, we observed a steady increase in the predicted adjusted incidence rate for 1 year heart failure hospitalization, almost doubling from 64.5 cases per 1000 person-years (95% CI: 51.1 to 78.0) in 2005, to 118.2 cases per 1000 person-years in 2019 (95% CI: 115.0 to 121.5). The differences in population demographics and study duration may partly explain the variations in secular trends regarding the new onset of HF. Additionally, prior to the implementation of revascularization strategies, such as the availability of primary PCI for STEMI and increased utilization of invasive approach in patients with NSTEMI, patients suffered greater myocardial damage resulting in subsequent HF. Therefore, studies including cohorts from the pre-PCI era are likely to show declining trends in the incidence of HF.^11^ Alternatively, the recent rise in HFH post-AMI may be due to improvement in revascularization thus leading to lower rates of competing outcomes such as sudden cardiac death or due to greater recognition and coding identification of HF. Nevertheless, our results confirm that the new onset of HF following AMI remains a significant problem in contemporary practice.

The poor prognosis of new onset of HF requiring hospitalization after AMI remains concerning, as it is marked by increased mortality rates. Kochar *et al.*^32^ reported a significantly higher 5-year mortality rate among patients with any heart failure post-MI compared to those without HF. Docherty *et al.*^11^ reported that annualized mortality was five-fold greater in those after a first hospitalization for HF compared to those without HF. Our study confirms the poor prognosis of HF complicating an AMI, as HFH was associated with a nearly three-fold increased hazard of 1-year mortality after adjusting for confounding factors. These findings underscore the persistent challenges in improving long-term outcomes for individuals with HF complicating AMI, emphasizing the need for continued efforts to increase uptake of guidelines-directed care during index hospital stay, timely reperfusion, and optimizing post-MI care strategies.^33,34^

In this study, we quantified the risk of HF hospitalization following AMI based on the number of risk factors at the time of index AMI as defined by the inclusion criteria of EMPACT-MI and DAPA-MI trials. Our results show a substantial increase in HFH, with over a quarter (25.7%) of patients with five or more risk factors being hospitalized within the 12-month follow-up. Similar, high hospitalization rates at 30 days and 1 year were observed in the DAPA-MI cohort. These data provide important insight into the early identification of patients at high risk of developing HF and subsequent hospitalization. These patients may benefit from optimizing risk factors, implementing newer HF therapies.^33,35,36^

Indeed, adherence to ESC quality indicators (QIs), such as timely reperfusion in STEMI and early invasive coronary angiography in NSTEMI patients, was associated with lower 1-year hospitalization rates in our analysis. Conversely, the prescription of HF disease-modifying agents such as beta-blockers, angiotensin channel inhibitors/receptor blockers, and mineral corticosteroid receptor antagonists at hospital discharge did not influence rates of subsequent HF hospitalization. Data from a recently published nationwide ACSIS survey also did not show any association between the number of HF medications prescribed and adverse clinical outcomes, including short-term HF hospitalizations.^37^ While a positive role of these secondary prevention medications in high-risk patients is well-established in the literature,^35,36,38–42^ the benefits may be attenuated in the post-AMI population in the current era. Findings from our study and similar registries^37,43^ could possibly be explained by the shortcomings associated with any observational analyses but mainly the lack of data on drug adherence and persistence.

Despite its strengths, including a multisource national cohort of AMI patients from the modern PCI era, and detailed in-hospital pharmacological and procedural management, several limitations may affect this report. Firstly, as the information is collected from existing records, the data quality is often variable and may contain inaccuracies. Secondly, we only included the first HF hospitalization following the index AMI admission, which might affect the overall burden, morbidity, and mortality associated with these unreported HF presentations. Although we were able to meet the majority of inclusion criteria for both EMPACT-MI and DAPA-MI trials, several key variables such as uric acid, pulmonary artery pressure and pro-BNP levels are not collected in the MINAP registry. However, as we observed a strong association between the cumulative number of risk factors as per inclusion criteria and subsequent HF hospitalization, we are likely to have underestimated the HF hospitalization rates. Myocardial Ischemia National Audit Project registry collects detailed information about the in-hospital and discharge pharmacology used for the treatment of ACS, including the use of beta-blockers, ACEI, and ARBs aldosterone antagonists and all models were adjusted for all available potential confounders. However, information about newer heart failure-related medications such as SGLT2, GLP-1, and ARNI information are not collected in the database which have limited our ability to fully adjust for the prognostic heart failure medications. Finally, the MINAP data registry shares the weaknesses of other national registries, including the self-reporting of adverse events with no external validation, potential biases, unmeasured confounding factors, and incomplete data, which may impact the generalizability and interpretation of our findings. MINAP does not collect data on cardiac imaging, which limits the ability to fully assess the impact of modern reperfusion therapies on post-AMI heart failure hospitalizations. Including imaging data in future analyses could offer valuable insights into the effectiveness of these treatments in mitigating heart failure following myocardial infarction.

## Conclusion

Despite advancements in pharmacological and revascularization strategies in the modern PCI era, a significant proportion of patients presenting with AMI suffer subsequent hospitalization with acute HF. Furthermore, increasing age, female sex, presence of LV dysfunction and number of risk factors for HF hospitalization defined by EMPACT-MI and DAPA-MI trials were associated with higher rates of 30-day and 1-year HF hospitalization. These findings underscore the importance of implementing targeted and comprehensive post-AMI care strategies involving a multidisciplinary approach, closer monitoring of high-risk patients, and tailored interventions to optimize post-AMI management, focusing on increasing the uptake of ESC quality of care indicators (QIs).

## Supplementary Material

oeaf013_Supplementary_Data

## Data Availability

The data underlying this article are available in the article and in its online supplementary material.
