# Supplementary material for: 15-Year trends, predictors, and outcomes of heart failure hospitalization complicating first acute myocardial infarction in the modern percutaneous coronary intervention era
Source: Eur Heart J Open. 2025 Feb 19;5(2):oeaf013. doi: 10.1093/ehjopen/oeaf013 (PMC11896973; doi:10.1093/ehjopen/oeaf013)
Supplement: oeaf013_Supplementary_Data [file oeaf013_supplementary_data.docx]

Supplementary Figure 1: Study flow diagram for final cohort derivation


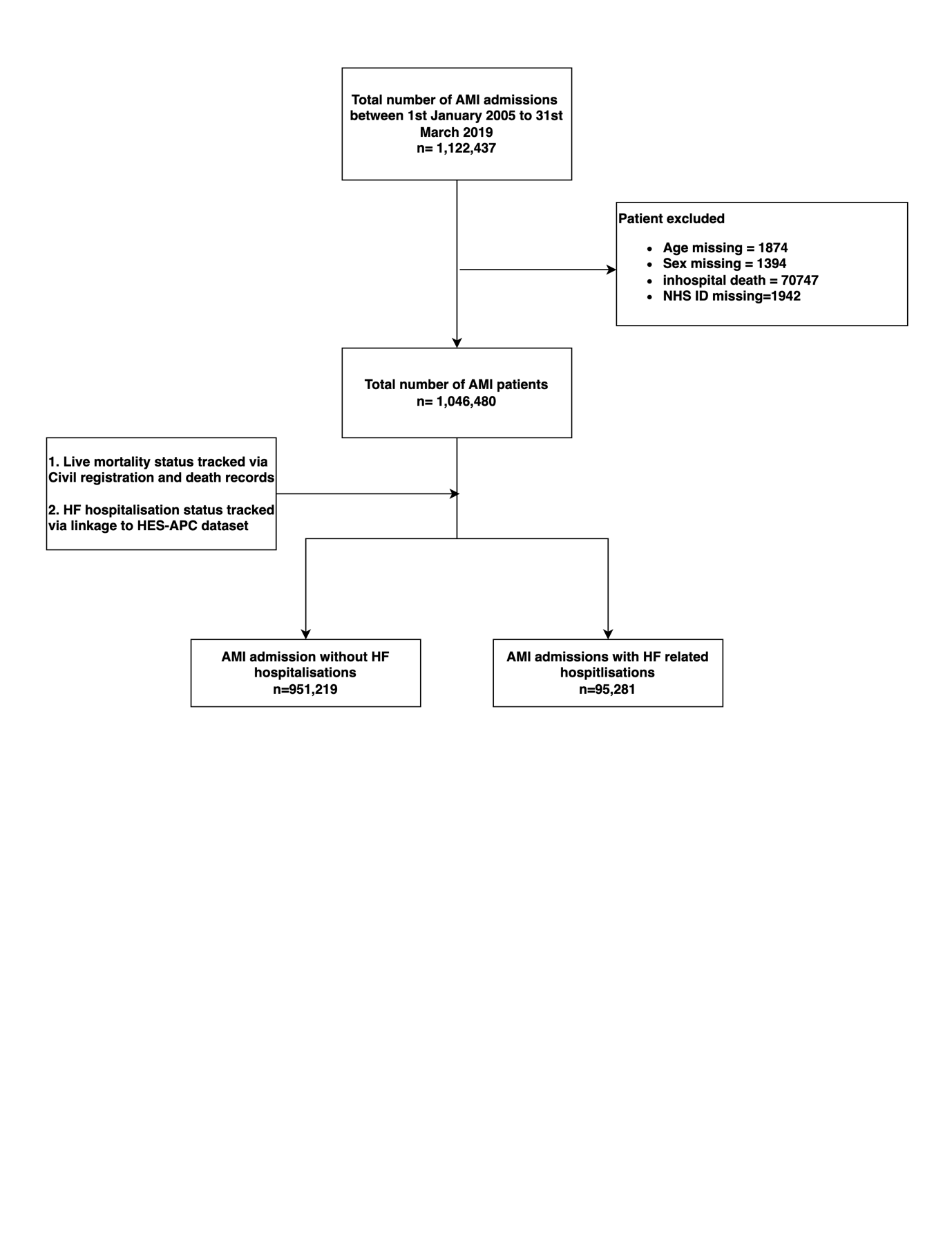


Supplementary figure 2: Temporal trends in rates of HF hospitalisations at 30 days and 1 year during the study period


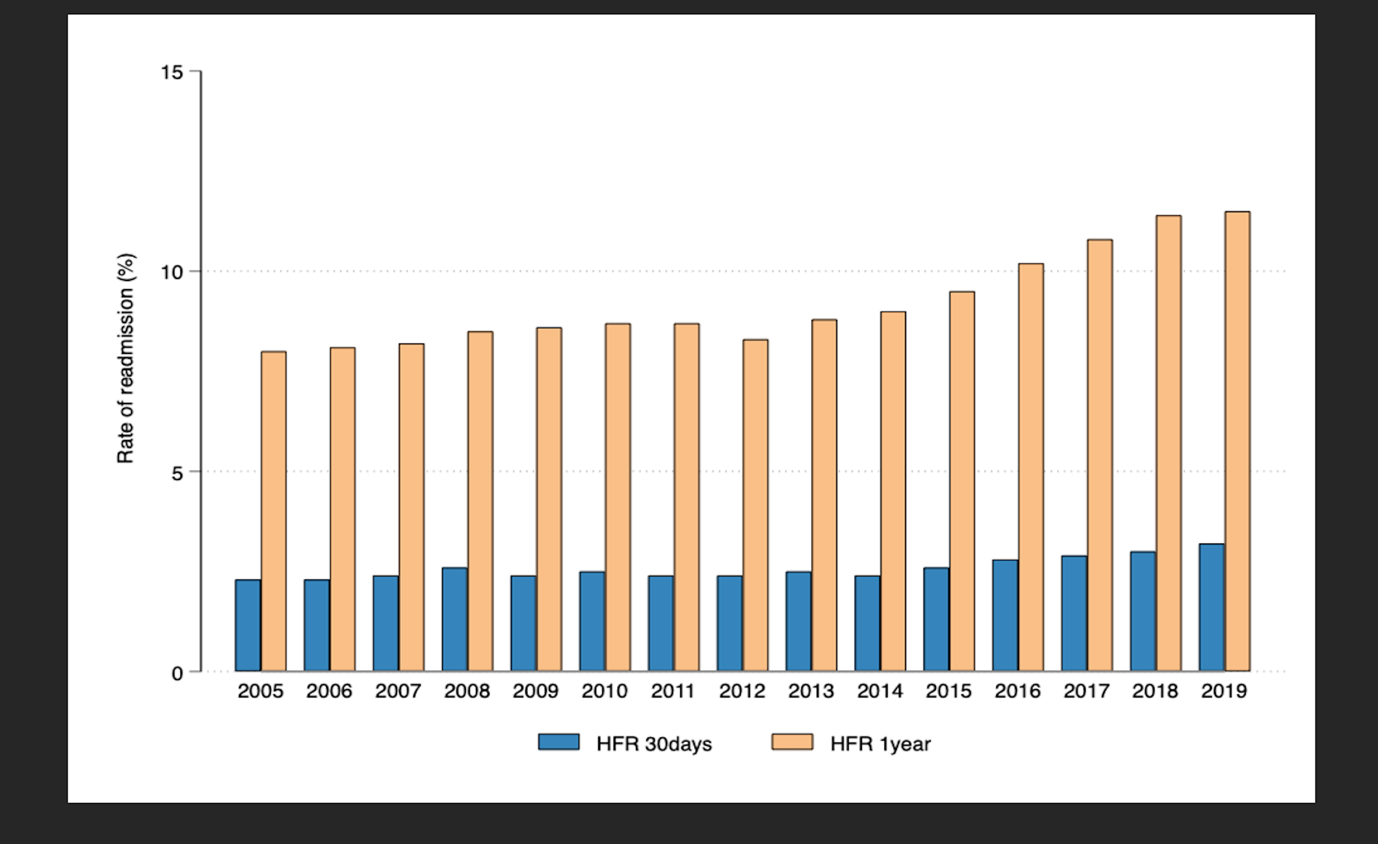


Supplementary Figure 3: Cumulative incidence of heart failure hospitalisation according to presence and absence of diabetes


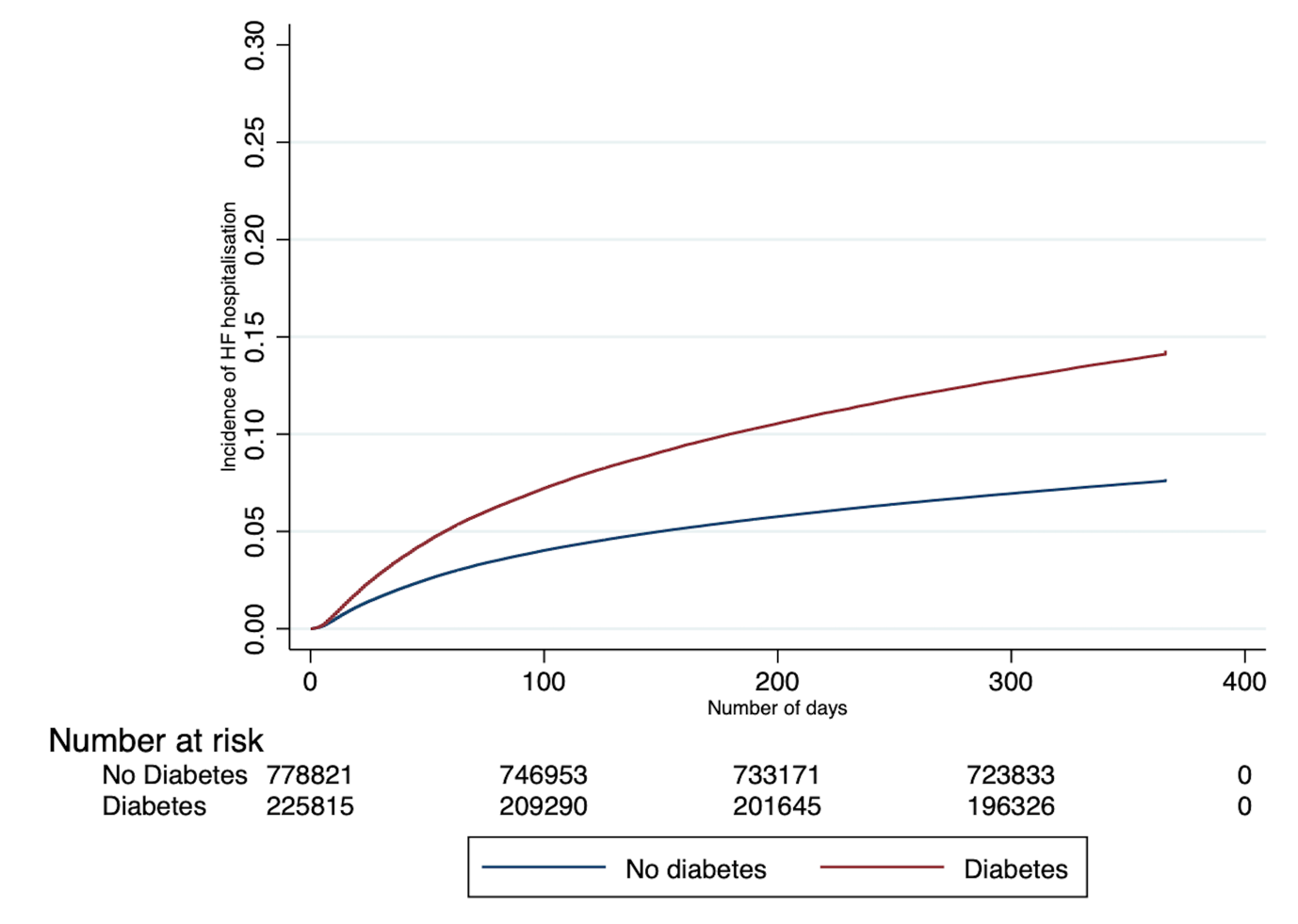


Supplementary Figure 4: Cumulative incidence of heart failure hospitalisation according to sex


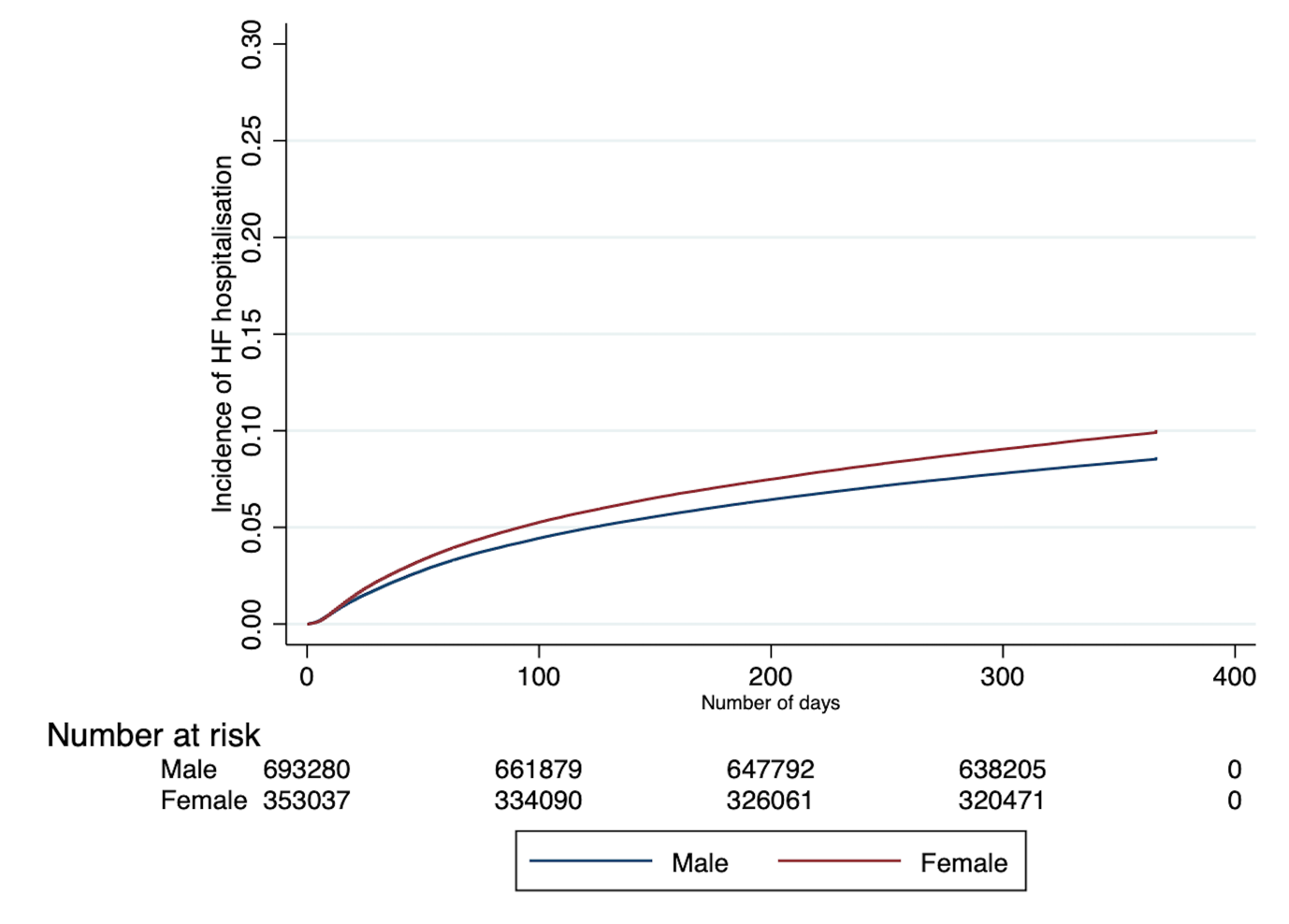


Supplementary Figure 5: cumulative incidence of heart failure hospitalisation according to age subgroups


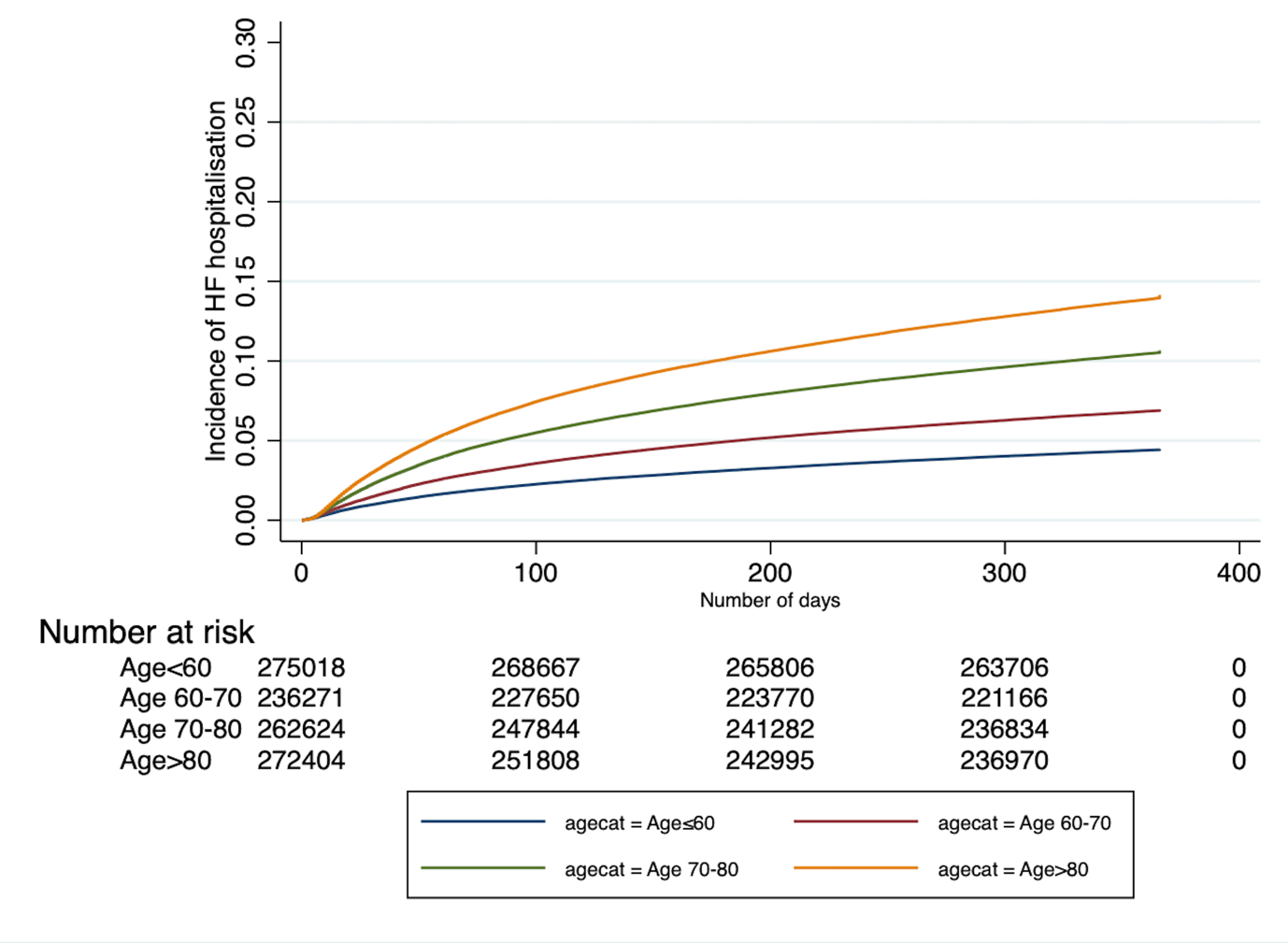


Supplementary table 1: Inclusion criteria for EMPACT-Mi and DAPA-Mi trials and definition from the MINAP registry

| **EMPACT-MI trial** | **MINAP registry criteria** |
| --- | --- |
| Diagnosis of acute spontaneous NSTEMI or STEMI | Discharge diagnosis from MINAP registry |
| Symptoms (eg, dyspnea, fatigue, decreased exercise tolerance) or signs of congestion (eg, pulmonary rales, crackles, crepitations, elevated jugular venous pressure, congestion on CXR) requiring treatment (augmentation or initiation of oral diuretic therapy, iv diuretic therapy, IV vasoactive agent, mechanical intervention) during hospitalization | Kilip Class 1-Kilip class IV, use of Thiazide or loop diuretics |
| Newly developed LVEF <45% as measured by echocardiography, ventriculography, cardiac CT, MRI, or radionuclide imaging during index hospitalization | Left ventricular ejection function (LVEF) defined in the registry |
| Age>65 | Age on admission |
| newly developed LVEF <35% | LVEF <35% |
| eGFR <60 ml/min/1.73 m2 | eGFR <60 ml/min/1.73 m2 |
| Atrial fibrillation | Use of Warfarin |
| Prior or new diagnosis of T2DM | Prior or new diagnosis of T2DM |
| NT-proBNP ≥1,400 pg/mL if in sinus rhythm, ≥2,800 pg/mL if in atrial fibrillation; | Not collected |
| Pulmonary artery systolic pressure (or right ventricular systolic pressure) ≥40mmHg (measured non-invasively, typically in clinically indicated post-MI echocardiography) or invasively, at any time during hospitalization | Not collected |
| Patient not revascularized (and no planned revascularization for index MI (includes patients with no angiography performed, unsuccessful revascularization attempts, diffuse atherosclerosis not amenable to intervention, but does NOT include patients for whom no revascularization was performed due to non-obstructive coronary artery disease). | Patient not receiving coronary angiography, or for medical management as collected in the registry |
| 3-vessel coronary artery disease at time of index MI | Not collected |
| diagnosis of peripheral artery disease | diagnosis of peripheral artery disease |
| **Inclusion criteria for DAPA-MI** | |
| Diagnosis of acute spontaneous NSTEMI or STEMI | Discharge diagnosis from MINAP registry |
| Imaging evidence of impaired regional or global LV systolic function at any timepoint during the index MI-related hospitalization (established with echocardiogram, radionuclide ventriculogram, contrast angiography or cardiac MRI) OR definitive evidence on ECG of a Q-wave MI (defined as presence of Q waves in two or more contiguous leads, excluding leads III and aVR, and meeting all the following criteria: at least 1.5 mm in depth; at least 30 ms in duration; and, if R wave present, more than 25% of the size of the subsequent R wave) | Left ventricular ejection function (LVEF) defined in the registry or any left ventricular dysfunction defined during the index hospital admission. |
